# The typhoon-induced drying of the Maritime Continent

**DOI:** 10.1073/pnas.1915364117

**Published:** 2020-02-10

**Authors:** Enrico Scoccimarro, Silvio Gualdi, Alessio Bellucci, Daniele Peano, Annalisa Cherchi, Gabriel A. Vecchi, Antonio Navarra

**Affiliations:** ^a^Fondazione Centro euro-Mediterraneo sui Cambiamenti Climatici, 40127 Bologna, Italy;; ^b^Istituto Nazionale di Geofisica e Vulcanologia, 40127 Bologna, Italy;; ^c^Department of Geosciences, Princeton University, Princeton, NJ 08544

**Keywords:** typhoon, maritime continent, precipitation, tropical cyclone

## Abstract

The Maritime Continent plays a role in the global circulation pattern, due to the energy released by convective condensation over the region which influences the global atmospheric circulation. We demonstrate that tropical cyclones contribute to drying the Maritime Continent atmosphere, influencing the definition of the onset of the dry season. The process was investigated using observational data and reanalysis. Our findings were confirmed by numerical experiments using low- and high-resolution versions of the CMCC-CM2 General Circulation Model contributing to the HighResMIP CMIP6 effort.

## Significance

Much work has been done to quantify tropical cyclone (TC)-induced precipitation and its role in determining flood events. We identify the role of tropical cyclones in reducing precipitation over particular areas of the tropical domain. The significant reduction in the precipitation over the Maritime Continent when the TC season is particularly active is in line with the net reduction in westward water flow into the Maritime Continent atmosphere, induced by TC-associated circulation over the region.

Several studies have analyzed the effects of mean climate conditions and climate change on tropical cyclone (TC) activity (1–11). There is increasing attention to the impact of TCs on the mean climate through their interaction with the ocean (12–16) and with the surrounding atmospheric environment. TC-induced stationary Rossby waves (17) are likely responsible for the interaction with the atmospheric environment, due to the fact that they excite extratropical wave trains affecting higher latitudes (18–22). Also, TC-associated water transport has a role in feeding extreme precipitation events in the extratropics (23, 24).

In this paper, we highlight the role of TCs as important players within Earth’s climate system (25). We evaluated the drying effect that TCs have on certain portions of the equatorial band, due to induced zonal wind anomalies. We found that a net eastward water transport anomaly in the equatorial region of the west North Pacific (WNP), induced by TCs developing in the basin, may be responsible for a significant moisture flux divergence over the Maritime Continent, thus reducing the local precipitation during the onset of the dry season. We investigated this process using Japanese 55-y Reanalysis (JRA-55) (26) and conducted numerical experiments based on low- and high-resolution versions of the Centro Euro-Mediterraneo per i Cambiamenti Climatici Climate Model 2 (CMCC-CM2) General Circulation Model (GCM) (16, 27, 28). Our findings suggest that forecasting TC activity in the WNP might also help in predicting the onset of the dry season over the Maritime Continent. This is based on the role of TCs in modulating the moisture flux over the region.

## TC Representation in Reanalysis and Climate Models

Climate modeling provides a realistic representation of TCs activity in terms of both their geographical density and intensity. GCMs (16, 29, 30) are now able to represent the most intense hurricanes and typhoons, mainly due to the horizontal resolution—equal or higher than 25 km—in their atmospheric component. Long reanalyses (such as JRA-55 used in the present work), based on GCMs, provide a considerable amount of climate information associated with the observed TCs (31, 32) at a high spatial and temporal resolution (a few hours). This detailed information associated with observed TCs, together with observations, goes back to 1979 and earlier, with sufficiently high spatial and temporal detail.

Reanalyses have played a key role in improving our knowledge of the TC−climate interaction, as demonstrated by studies on typhoon-associated changes in the atmospheric dynamics (33, 34) or on induced TC modulation of the Arctic sea ice (22), and surface (35) and subsurface (36, 37) ocean temperatures.

In contrast with reanalyses, GCM numerical experiments analyze TC statistics under simplified experimental settings. These include “control” simulations performed using greenhouse gas (and aerosol) concentrations held fixed at conditions such as preindustrial or present climate. In the context of TC-focused analyses, control simulations are particularly suitable for isolating natural variability from human-induced changes.

The use of different horizontal resolutions in the atmospheric component of a fully coupled GCM (such as CMCC-CM2) helps to highlight the role of TCs within the climate system. Our model realistically represents the mean climate conditions in both its low- (CMCC-CM2-HR) and high-resolution (CMCC-CM2-VHR) configurations (the acronyms are the same as those used in the Climate Model Intercomparison Project 6 [CMIP6] framework to distinguish the different versions of our model). However, a horizontal resolution coarser than 100 km (used here by the CMCC-CM2-HR model and similar to those used by CMIP5 generation models) does not resolve intense hurricanes and typhoons: Only a few weak TCs are simulated. On the other hand, a 25-km-resolution atmospheric component (adopted here in the CMCC-CM2-VHR model) is able to represent the most intense hurricanes and typhoons.

A comparison of TC-associated precipitation, between the two versions of the model, can shed light on the role of the hurricane/typhoon in modulating atmospheric water transport dynamics. As a measure of the different abilities of low (HR) and high resolutions (VHR) in representing TCs over the west North Pacific, *SI Appendix*, Fig. S1 shows how the HR model generates fewer TCs compared to the realistic representation provided by VHR, in terms of both number and intensity.

We measured the energy dissipated by TCs over a region in a particular period through the accumulated cyclone energy (ACE) (38), which accounts for storm duration, intensity, and count, but not for cyclone size. The ACE is defined as the squared wind speed of each TC active in the considered region accumulated every 6 h. The WNP ACE is well modeled by the high-resolution VHR model in terms of both long-term averages and interannual variability, whereas the low-resolution HR model shows ACE values of one order of magnitude lower than the observed ones. These two versions of the CMCC-CM2 GCM are used to support our findings in the following section.

## TC-Induced Drying of the Maritime Continent: The Mechanism

**Fig. 1.**
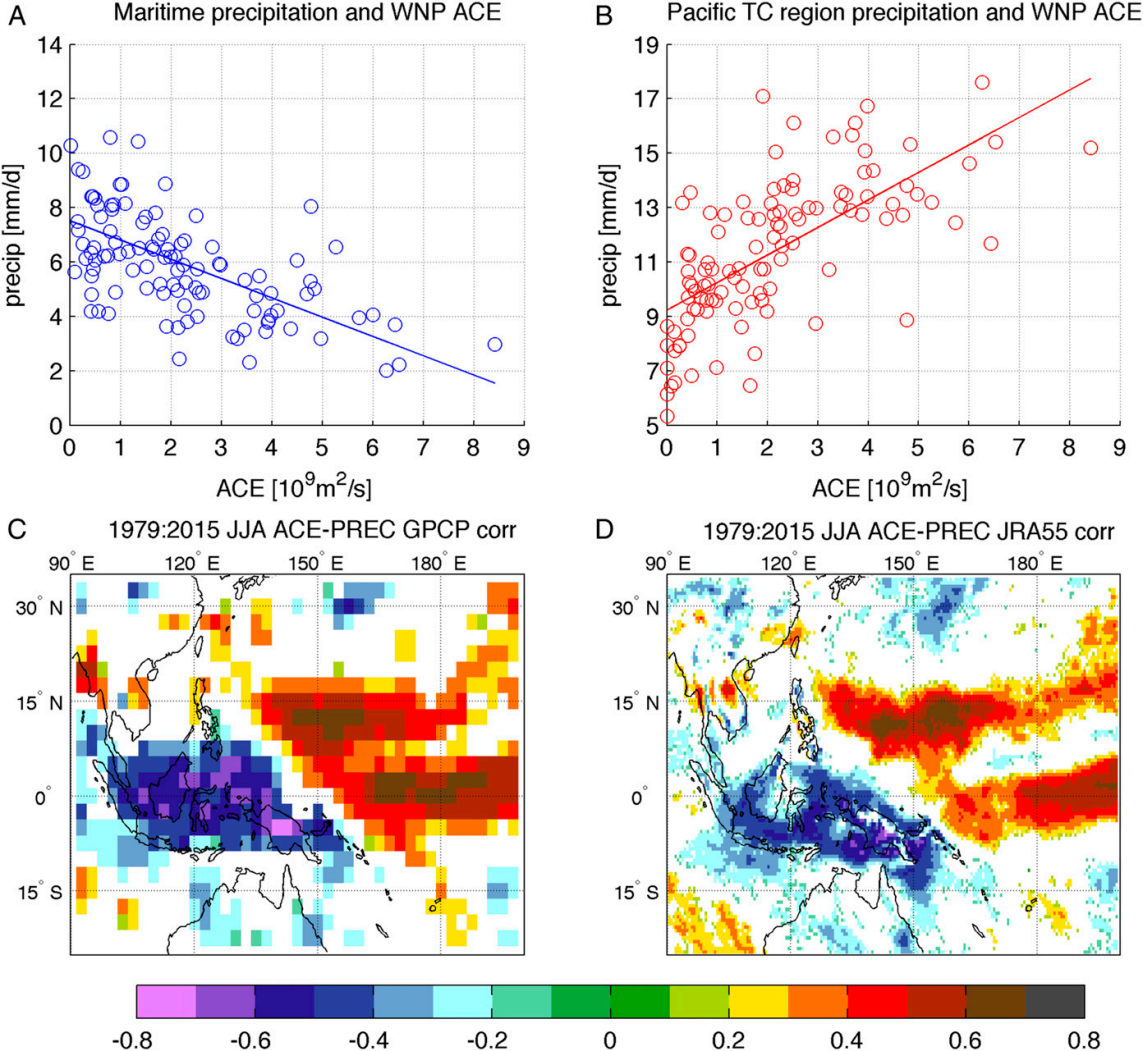
Correlation of precipitation with ACE. (*A* and *B*) Scatter plots of JJA monthly values of ACE over the WNP (units are 10^9^ square meters per second) and GPCP precipitation (units are millimeters per day) over (*A*) the Maritime Continent (8°S to 8°N, 100°E to 135°E) and (*B*) the West Pacific TC region (8°N to 22°N, 120°E to 155°E) from 1979 to 2015. (*C* and *D*) The correlation between ACE and the GPCP/JRA-55 precipitation for the same period, with average JJA values. White patterns represent regions where the correlation is not statistically significant.

The ACE averaged over the WNP is significantly and positively correlated with the precipitation in the West Pacific TC region (Fig. 1*B*). This is because TCs contribute significantly to the precipitation at these latitudes in the corresponding months (39) from June to August (JJA, for the period 1979–2015). Data from both the Global Precipitation Climatology Project (GPCP) (40) and JRA-55 reanalysis confirm this contribution (Fig. 1 *B*–*D*). At the same time, a strong and statistically significant negative correlation (lower than −0.7) appears at lower latitudes (Fig. 1 *A*, *C*, and *D*), suggesting a link between the WNP TC activity and the precipitation over the Maritime Continent.

**Fig. 2.**
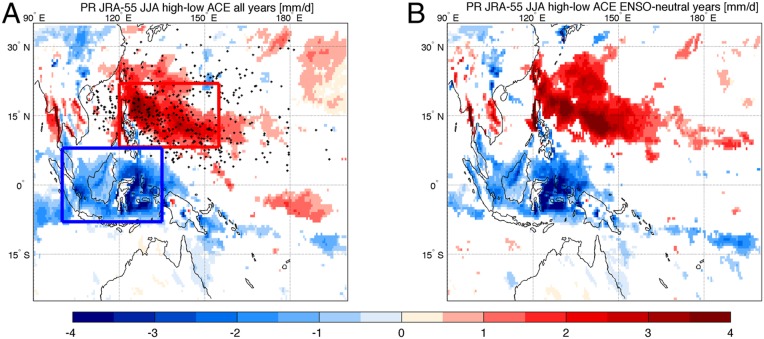
Comparison between precipitation associated with high-ACE and low-ACE years. The difference between JJA JRA-55 precipitation associated with high-ACE (higher than the median) and low-ACE years is shown (*A*) considering the whole period 1979–2015 and (*B*) considering ENSO neutral years only. Units are millimeters per day. Black dots represent the origin of TCs observed in this period. Blue and red boxes in *A* mark the domain used to compute the Maritime Continent and West Pacific TC region averages, respectively. White patterns represent regions where the difference is not statistically significant.

Similar patterns are obtained by computing the difference between the JJA precipitation averaged over the years with “high” (higher than the median) and “low” (lower than the median) ACE values (Fig. 2*A*). In line with this, similar patterns appear for the differences in precipitation between active TC days and inactive TC days within a single JJA season (results for 2002 are shown in *SI Appendix*, Fig. S2*H*).

The main external factors potentially interacting with both WNP TC activity and Maritime Continent precipitation are the El Niño−Southern Oscillation (ENSO) (41), the Madden and Julian Oscillation (MJO) (42), the Pacific−Japan pattern (PJ) (43), and the oscillation of the Hadley circulation in terms of both meridional position and intensity (44).

In order to evaluate the potential external influences of ENSO in modulating both the TC activity and the precipitation over the Maritime Continent, thereby determining their correlation, we stratified the precipitation patterns based on positive, neutral, and negative ENSO events (see *Methods* for details). The difference between the precipitation associated with high- and low-ACE years (Fig. 2*A*) is maintained also when considering ENSO neutral years only—about 20 y out of the total 37 y considered (Fig. 2*B*). In addition, in order to verify the potential role of MJO, PJ, and the Hadley circulation position and intensity (see *Methods*) in determining the correlation between TC activity and the Maritime Continent precipitation, we verified that, when considering MJO inactive years alone, or positive and negative phases of PJ and Hadley cell indices, there was still a significantly higher precipitation associated with inactive TC years (*SI Appendix*, Fig. S2).

**Fig. 3.**
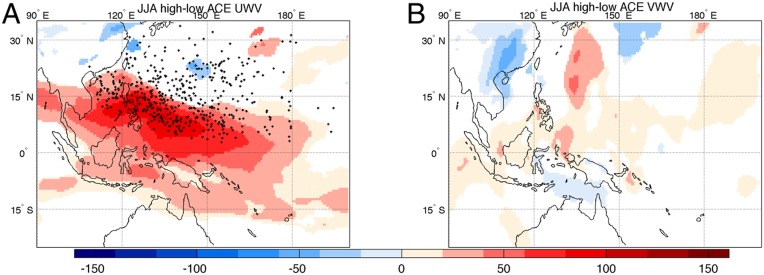
Comparison between vertically integrated water transport associated with high-ACE and low-ACE years. The difference between JJA JRA-55 vertically integrated (*A*) zonal and (*B*) meridional water transport associated with high-ACE (higher than the median) and low-ACE years is shown, considering the whole period 1979–2015. Units are kilograms per meter per second. Black dots represent the origin of TCs observed in this period. White patterns represent regions where the difference is not statistically significant.

A possible explanation for such a significant negative correlation between the WNP ACE and precipitation over the Maritime Continent lies in the composite effect that TCs can have in causing eastward wind anomalies at low latitudes where the TC-associated winds still have a significant magnitude (13). These TC-induced eastward wind anomalies, which are more pronounced during high-ACE years, contribute to the eastward transport of vertically integrated water content (Fig. 3*A*) away from the Maritime Continent domain. When the TC season is particularly active, there is thus less water available for local precipitation.

**Fig. 4.**
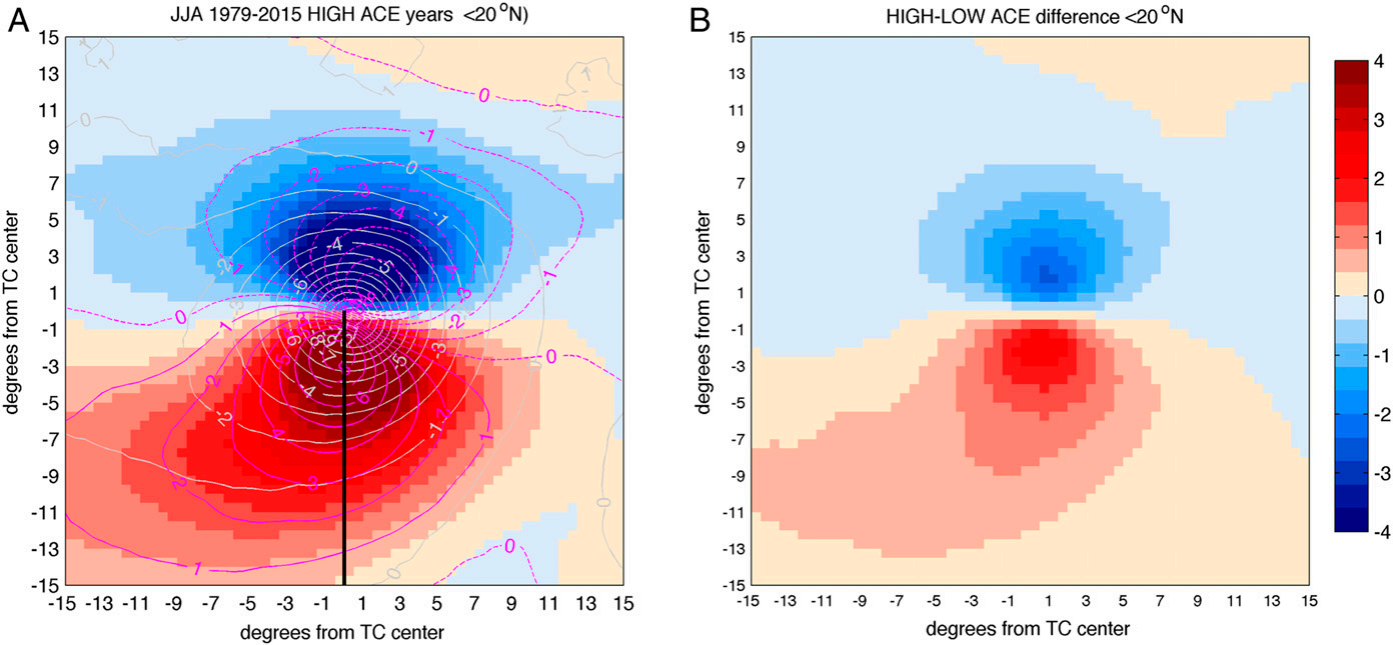
(*A*) JJA TC daily composite anomalies with respect to the daily 1979–2015 climatology during HIGH ACE years and (*B*) difference between HIGH and LOW ACE years over WNP. TC accumulated zonal water transport (color shading, 10^10^ kilograms per meter, positive values indicate eastward transport), zonal wind velocity (purple lines, meters per second), and sea level pressure (light gray lines, hectopascals) anomalies are shown. Only TCs traveling south of 20°N are considered. *B* shows the difference between HIGH and LOW composite accumulated water transport over the 1979–2015 period (units are 10^10^ kilograms per meter). The black line in *A* indicates the section used to integrate TC-associated eastward water transport.

Fig. 4 shows a quantification of the vertically integrated transport associated with TCs traveling south of 20°N, thus potentially affecting the Maritime Continent. The total amount of water transported eastward by TCs during high-ACE years is represented by red patterns in Fig. 4*A*, and the difference between high- and low-ACE years is shown in Fig. 4*B*. The total amount of water transported eastward during high-ACE years, integrated from the TC center to 15° south of the TC center (black line in Fig. 4*A* indicates the section used to integrate TC-associated eastward water transport), thus affecting Maritime Continent latitudes, is 60% higher than the same value accumulated during low-ACE years. This increases to 75% when considering TCs traveling south of 15°N only (not shown). The described increase in the total amount of water transported eastward by TCs during high-ACE years is consistent with the differences found in the averaged eastward transport over the region (Table 1, leftmost column).

**Table 1.** Integrated flows during JJA over the Maritime Continent box (8°S:8°N to 100°E:135°E box; see Fig. 2*A*) across its boundaries (east, west, north, south, surface) based on JRA-55 reanalysis data

	Flow boundary east	Flow boundary west	Flow boundary north	Flow boundary south	Downward flow (P-E)
HIGH ACE	−1,002.7	2.9	3,073.7	−1,916.6	121.3
LOW ACE	−2,037.5	236.2	2,767.3	−1,887.9	1,135.7
HIGH−LOW	1,034.8	−233.3	306.4	−28.7	−1,014.4

Composite over high- and low-ACE years are shown in the first and second rows, respectively, while the third row contains the difference between the two. The vertical extent considered for the integration reaches the top of the modeled atmosphere. Positive values refer to flows leaving the box. Units are 10^12^ kilograms. P-E, precipitation − evaporation.

In order to evaluate and quantify this effect of TC-induced water transport on the Maritime domain, we computed the water flow through each surface of the “Maritime Continent box.”

The large reduction in the net precipitation over the Maritime Continent during high-ACE years compared to low-ACE years (Table 1, rightmost column) is consistent with the net reduction in westward water flow into the Maritime Continent atmosphere. In fact, a more pronounced positive anomaly was found—reducing the westward flux—over the eastern bound during high-ACE years, compared to low-ACE years (Fig. 3*A*). This finding is also in agreement with previous results (45) highlighting negative precipitation anomalies in the area 5° to 20° apart from the TC center at west and south quadrants of TC in the WNP.

Many factors affect the interannual variability of precipitation over the Maritime Continent, and ENSO is considered the key player, especially during the dry season. However, our findings suggest that interannual variability in TC activity also plays a role. In order to demonstrate the role of TCs in drying the Maritime Continent, we also exploited GCM experiments. Although the CMCC-CM2 fully coupled model demonstrates a reasonable representation of the tropical mean climate and variability in both low (HR 110 km, CMIP5-like horizontal resolution) and high (28, 16) [VHR 25 km, HighResMIP CMIP6-like resolution (46, 47)] resolution, the main difference between the two is the weakness of the lower-resolution model in representing the number and intensity of TCs (*SI Appendix*, Fig. S1).

The observed reduction in precipitation over the Maritime Continent associated with particularly active TC years, induced by the modeled TC-induced water transport (*SI Appendix*, Figs. S3 and S4), is reasonably well represented only in the high-resolution VHR simulation (see *SI Appendix*, Fig. S5*B* compared to the observed results in Fig. 2), where TCs are well represented. On the other hand, the lower-resolution HR simulation is not able to reproduce the process that is investigated in the present study (*SI Appendix*, Figs. S4 and S5*A*). In fact, no significant signal was found in this case. Model results confirmed our hypothesis regarding the TC-induced effect in drying the Maritime Continent and modulating the Maritime Continent precipitation. This is shown by the modeled TC-induced vertically integrated water transport, which is only evident and significant in the high-resolution VHR simulation. In order to further corroborate our findings, we performed the following additional simulation: Using the VHR model configuration, we rerun the specific month, within the 30-y simulation, characterized by the highest ACE but forcing to zero the evaporation flux over the TC development region (8°N:30°N to 120°E:180°E) so as to inhibit the TC formation. *SI Appendix*, Fig. S6 shows the monthly mean difference between the original (unperturbed) and the TC-inhibited runs in terms of sea level pressure (contours) and precipitation (shading). The difference patterns reveal 1) a sea level pressure reduction and precipitation increase over the TC development region and 2) a precipitation reduction over the Maritime Continent. This is consistent with the hypothesized influence of TCs on Maritime Continent precipitation.

## Implications for the Forecast of the Dry Season Onset

TCs over the WNP start to develop during the wet to dry transition (May to June) (48–52) of the Maritime Continent. The present study highlights and quantifies the TC contribution to the drying of the Maritime Continent atmosphere (see previous section), thus playing a role in defining the onset and duration of the dry season.

The well-known tendency of the onset of the Maritime Continent dry season to develop earlier during El Niño conditions is also consistent with the role played by the expected increase in TC activity (38). Also, the tendency of the onset to develop later during La Niña conditions is in agreement with the expected decrease in TC activity. However, WNP TCs also modulate the amount of water available for precipitation over the region (Fig. 3) during non-ENSO years. This is also evidenced by the higher ACE values associated with low Maritime precipitation periods and by the higher Maritime precipitation associated with low-ACE periods (*SI Appendix*, Fig. S7) when considering the entire Maritime Continent box: The TC-induced change in precipitation (HIGH−LOW ACE years) amounts to 25% and 20% of the JJA and annual precipitation climatology, respectively. For this reason, forecasting TC activity months in advance (53, 54) over the WNP may help in forecasting the onset and duration of the dry season over the Maritime Continent. Consequently, forecasting TC activity can help improve, at least partially, the forecasts for all of the processes associated with the Maritime Continent circulation (55–60).

The WNP is where TC activity is at its highest and also the TC peripheral area is large, consistent with the high number of TCs reaching the largest size (61). For these reasons, the induced drying effect is expected to be more pronounced over the WNP compared to other regions.

These results are valid based on our ruling out a number of potential major third-party causal drivers, including ENSO, MJO PJ, oscillation of the Hadley cell, and the consistency between TC eastward water vapor transport and the concurrent precipitation decrease over the Maritime Continent. All of the above processes and phenomena are embedded within the tropical circulation, making it hard to identify a unique cause−effect mechanism. We cannot exclude that there may be other potential mechanisms at play here that we have not considered, and future work may investigate a broader set of drivers beyond what is done here. In addition, future work will follow the methods described here to explore whether the highlighted TC-induced drying over the Maritime Continent might also apply to cyclones in other basins.

## Methods

To compute the monthly observed WNP ACE from 1979 to 2015, we used TC best-track data from the Japan Meteorological Agency Regional Specialized Meteorological Center Tokyo. The environmental conditions associated with the different ACE conditions observed were inspected based on JRA-55 reanalysis, and, for a more extensive description of this dataset, the reader is referred to ref. 26.

Modeling analysis was based on CMCC-CM2 (28) HR (100 km) and VHR (25 km) results, obtained following 1950 perpetual radiative forcing conditions for a period of 30 y. Our modeled TC-associated ACE was based on TC tracks obtained following the procedure described in ref. 16 to CMCC-CM2 model output: Potential TC conditions are identified based on 6-hourly 850-hPa relative vorticity, maximum wind, local sea level pressure, and warm core (based on temperature averaged between 300 and 500 hPa). Then, TCs are tracked, verifying, for each potential TC condition, the presence of TCs during the following 6-h time period within a distance of 400 km. If no TC condition is found, the trajectory is considered finished. A tracked trajectory is qualified as a TC if it lasts at least 3 d.

For the definition of the HIGH−LOW ACE conditions, we first computed the median of the 1979–2015 observed and modeled JJA ACE annual time series. Then we computed the difference between the averaged values associated with ACE years higher than the median anNd those lower than the median. For the definition of the HIGH−LOW conditions, there are no significant differences considering detrended and not detrended ACE time series.

Positive, neutral, and negative ENSO phases were identified based on sea surface temperature anomaly over the nino3 (5°N to 5°S, 150°W to 90°W) region: A temperature higher than 0.5 °C identifies a positive phase, a temperature between −0.5 °C and +0.5 °C identifies a neutral phase, and a temperature lower than −0.5 °C identifies a negative phase.

MJO inactive years were selected based on National Oceanic and Atmospheric Administration (NOAA) data (https://www.cpc.ncep.noaa.gov/products/precip/CWlink/daily_mjo_index/proj_norm_order.ascii) providing MJO index time series also for region 3 (the one corresponding to 120°E within the 10 available). We selected MJO inactive years based on JJA values (computed starting from 5-d time series provided by NOAA) lower than 0.5. Positive and negative phases of the PJ pattern were obtained based on ref. 43, and indices for Hadley circulation Hedge Intersect position (HCE_I) and Intensity in the Northern Hemisphere (HCI_N) were computed based on JRA-55 reanalysis data following ref. 44.

The statistical significance of the differences and of the Pearson correlation coefficients referred to in this paper was verified at the 95% level with a bootstrap method. The correlation coefficients were computed after linear detrending of the considered time series.

We also integrated the water fluxes that affect the Maritime Continent box and West Pacific TC region encompassing 8°S:8°N to 100°E:135°E and 8°N:22°N to 120°E:155°E, respectively, as highlighted in Fig. 2*A* (blue and red rectangles), for JJA. Table 1 shows the integrated flows along the surfaces of the Maritime Continent box (excluding the upper surface) in terms of composites during high- and low-ACE years. The same regions were used for the computation of precipitation averages considered in Fig. 1 *A* and *B* and, additionally, in *SI Appendix*, Figs. S2 and S7. The data analyzed in this paper are available at https://esgf-node.llnl.gov/search/cmip6/ or upon request from the corresponding author.

## Supplementary Material

Supplementary File
