# Supplementary material for: The typhoon-induced drying of the Maritime Continent
Source: Proc Natl Acad Sci U S A. 2020 Feb 10;117(8):3983–8. doi: 10.1073/pnas.1915364117 (PMC7049124; doi:10.1073/pnas.1915364117)
Supplement: Supplementary File [file pnas.1915364117.sapp.pdf]

The typhoon-induced drying of the Maritime Continent

Enrico Scoccimarro[1*], Silvio Gualdi[1], Alessio Bellucci[1], Daniele Peano[1], Annalisa Cherchi[2], Gabriel A. Vecchi[3] and Antonio Navarra[1]

[1] Fondazione Centro euro-Mediterraneo sui Cambiamenti Climatici - CMCC, Bologna, Italy.
[2] Istituto Nazionale di Geofisica e Vulcanologia - INGV, Bologna, Italy.
[3] Princeton University - Princeton, New Jersey.

[*]e-mail: enrico.scoccimarro@cmcc.it

Supplementary Tables and Figures

www.pnas.org/cgi/doi/10.1073/pnas.1915364117

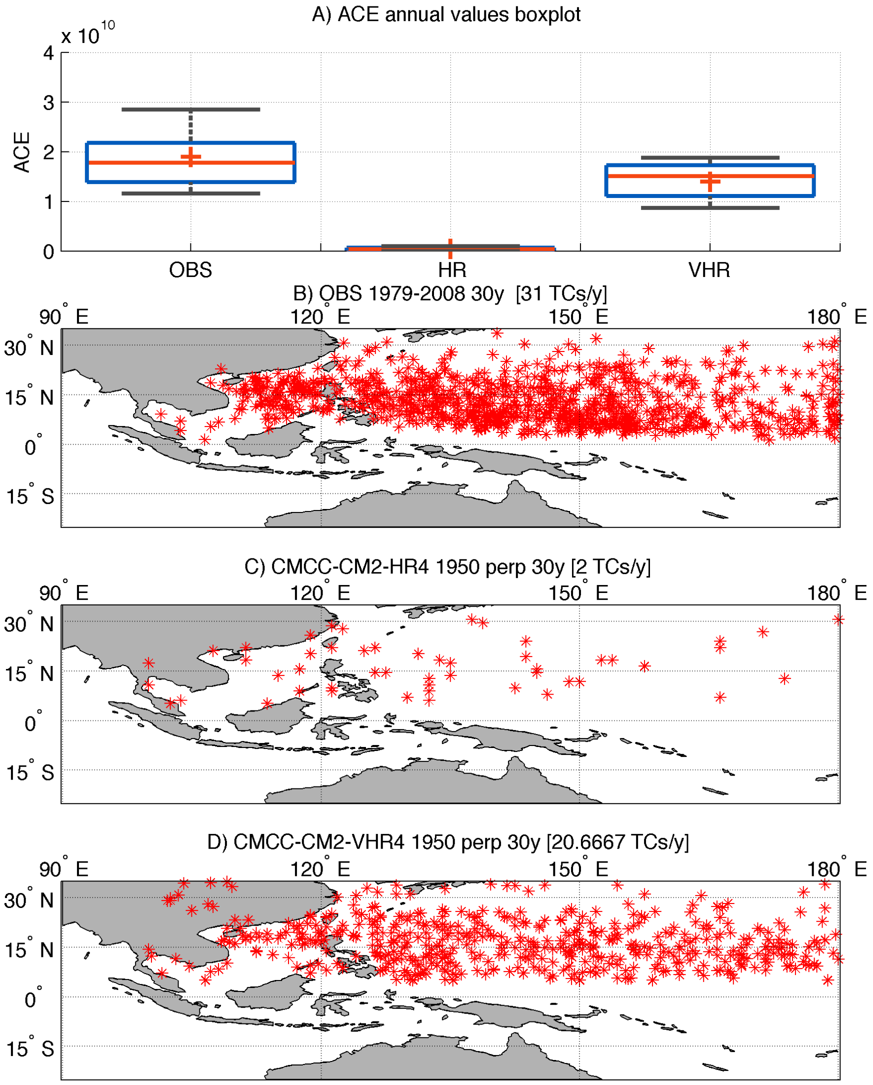

Figure S1. Tropical cyclone representation in CMCC-CM2 model. Upper panel shows the box plot of annual Accumulated Cyclone Energy over the West North Pacific basin, considering 30 years, for observations, CMCC-CM2-HR, CMCC-CM2-VHR in left, central and right boxes respectively. Units are [m^2/s]. Panels B, C and D show the geographical distribution of observed and modelled TC genesis location in the 30 year period.

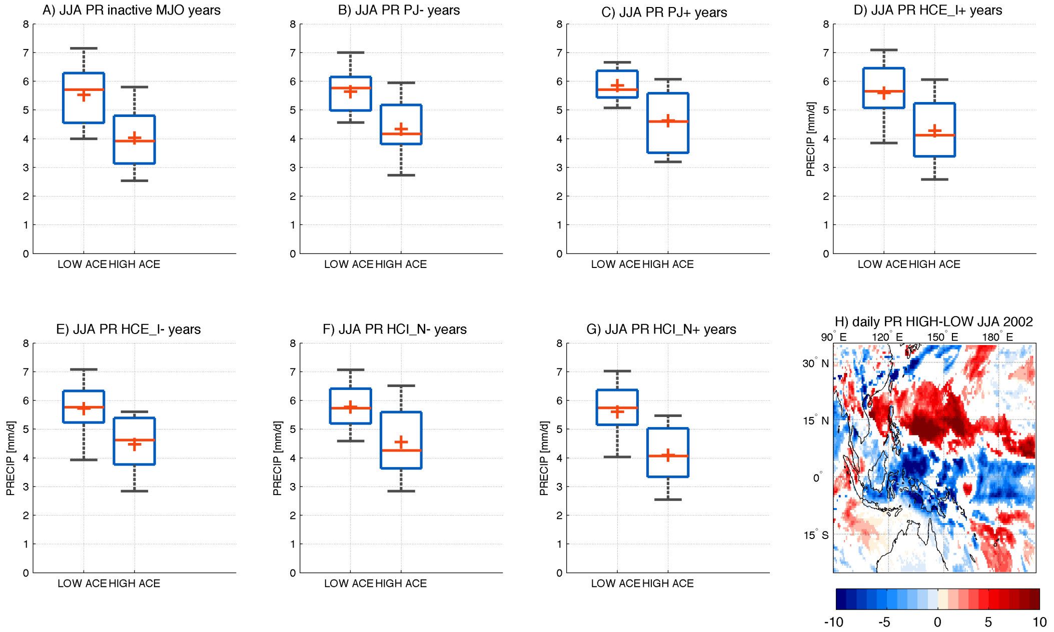

Figure S2. Observed Maritime precipitation during high and low ACE years. Observed averaged precipitations over the Maritime Continent (considering the "Maritime Continent box" encompassing 8°S:8°N-100°E:135°E) during JJA, derived from stratifying over low (LOW ACE) and high (HIGH ACE) ACE years from 1979 to 2015. Box plots were computed considering inactive MJO years only (panel A), negative phase of the Pacific Japan pattern (panel B), positive phase of the Pacific Japan pattern (panel C), positive phase of the Hadley Cell Interesect Index (panel D), negative phase of the Hadley Cell Interesect Index (panel E), negative phase of the northern Hadley Cell Intensity index, (panel F), ND positive phase of the northern Hadley Cell Intensity index, (panel G). The Maritime precipitation was averaged considering years with lower/higher ACE than the climatological monthly value (LOW ACE / HIGH ACE). Panel H) shows the difference between precipitation averaged during TC active days and non active TC days in a single JJA season (during the 2002 year). Units are [mm/d].

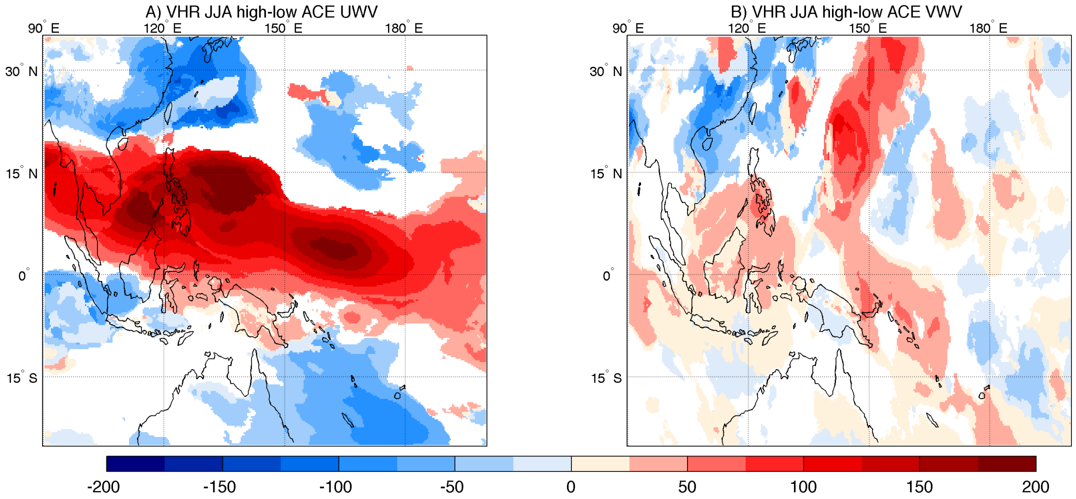

Figure S3. Same as Figure 3 but derived from the high resolution CMCC-CM2-VHR4 model: Comparison between vertically integrated water transport associated with high-ACE and low-ACE years. The difference between JJA CMCC-CM2-VHR4 vertically integrated zonal (panel A) and meridional (panel B) water transport associated with high-ACE (higher than the median) and low-ACE years is shown considering the entire 30 years period. Units are [Kgm^{-1}s^{-1}]. White patterns represent regions where the difference is not statistically significant.

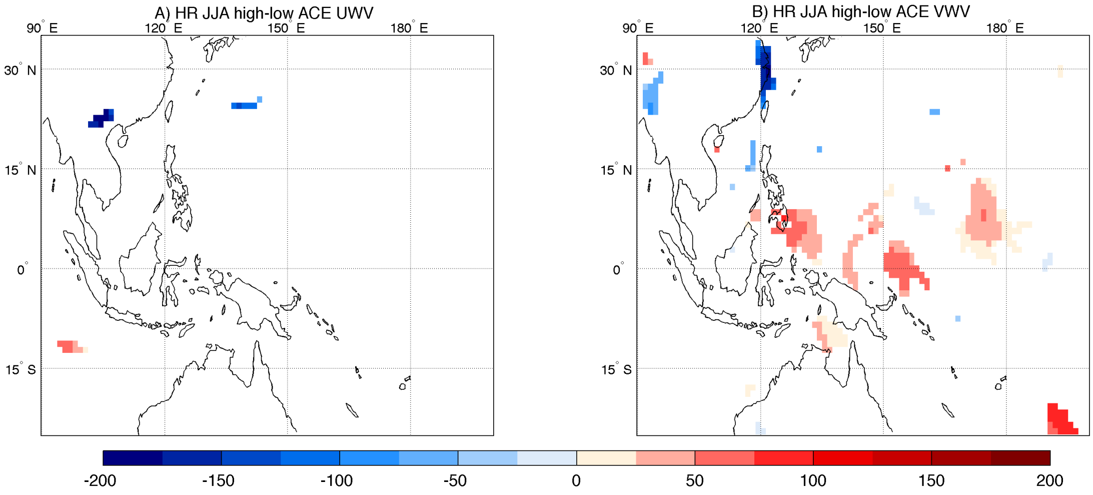

Figure S4. Same as Figure 3 but derived from the low resolution CMCC-CM2-HR4 model.

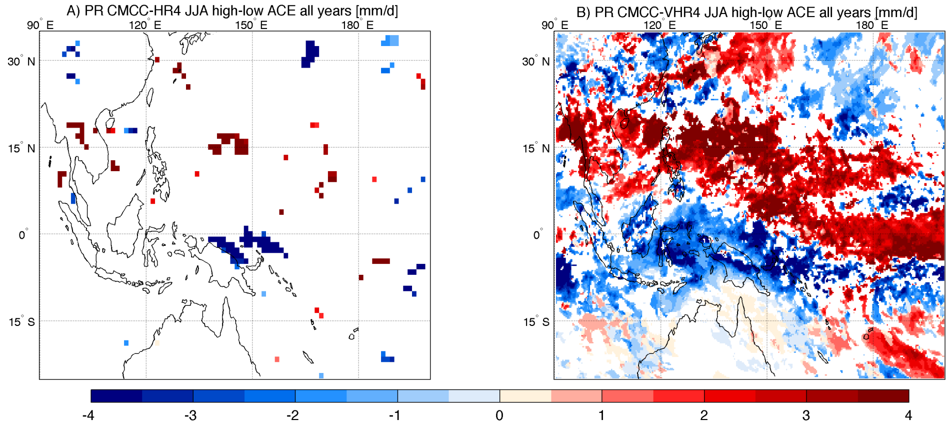

Figure S5. Comparison between precipitation associated with high-ACE and low-ACE years from the models. Same as Figure 2 but computed based on the results of the model. The difference between precipitation associated with high-ACE (higher than the median) and low-ACE years derived from the low- and high- resolution versions of the CMCC-CM2 model are shown in panels A) and B) respectively, considering a 30-year period. Units are [mm/d]. White patterns represent regions where the difference is not statistically significant.

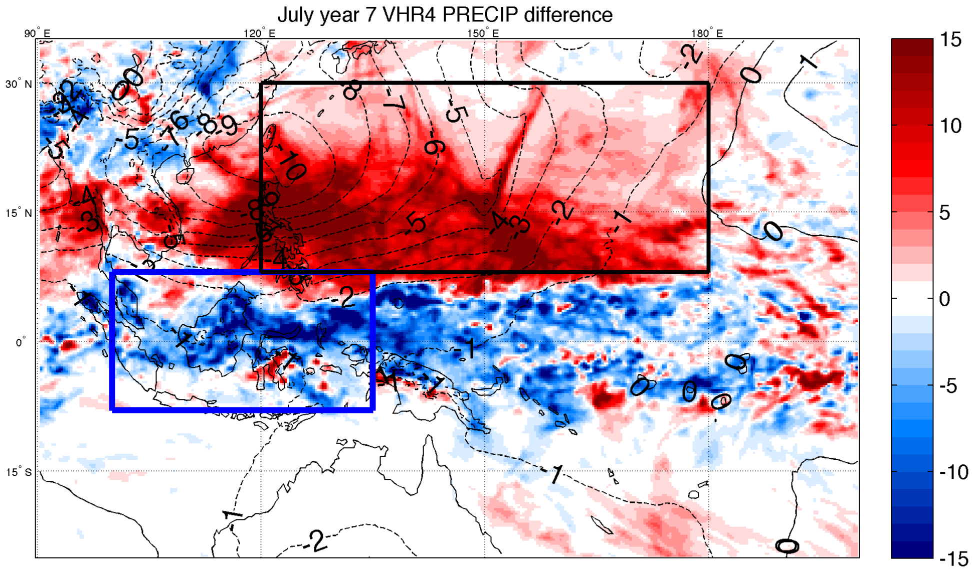

Figure S6. Precipitation and sea level pressure difference in a CMCC-CM2-VHR4 simulation with inhibited TC development compared to the standard run: the CMCC-CM2_VHR4 simulated month corresponding to the highest ACE (July of year 7) within the considered season has been rerun starting from the same initial conditions but forcing the evaporation to zero over the TC development box (8°N:30°N-120°E:180°E, black box) to inhibit TC development. The differences between the original simulation and the simulation with TC development inhibited, are shown. Precipitation units are [mm/d]. Black contours represent sea level pressure difference (dashed lines for negative values). Sea level pressure units are [hPa]. Blue box is the same shown in figure 2 to encompass the Maritime Continent.

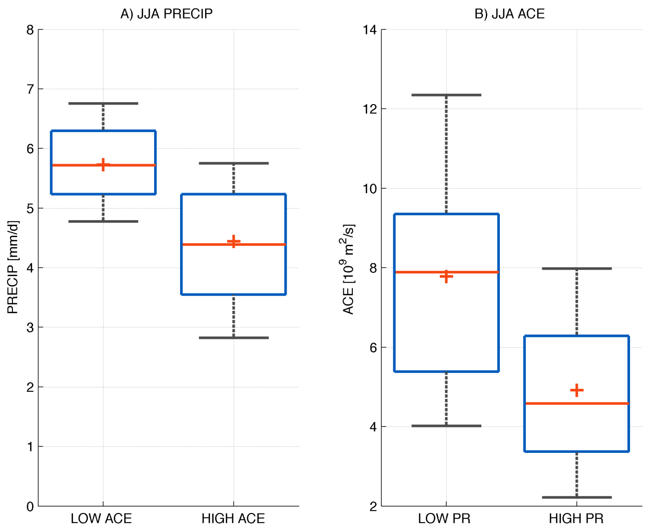

Figure S7. Observed Maritime precipitation during high and low ACE years and observed ACE during high and low Maritime precipitation years and. Panel A) shows the observed averaged precipitations over the Maritime Continent during JJA, derived from stratifying over low (LOW ACE) and high (HIGH ACE) ACE years from 1979 to 2015. Units are [mm/d]. The Maritime precipitation was averaged considering years with lower/higher ACE than the climatological monthly value (LOW ACE / HIGH ACE). Panel B) shows the observed annual Accumulated Cyclone Energy over the West North Pacific basin during JJA derived from stratifying over low (LOW PR) and high (HIGH PR) Maritime precipitation years from 1979 to 2015. Units are [$10^9 m^2/s$]. ACE was averaged considering years with Maritime precipitations lower/higher than the climatological monthly value (LOW PR / HIGH PR).
